# Supplementary figures and images for: Spatiotemporal dynamics of human high gamma discriminate naturalistic behavioral states
Source: PLoS Comput Biol. 2022 Aug 8;18(8):e1010401. doi: 10.1371/journal.pcbi.1010401 (PMC9387937; doi:10.1371/journal.pcbi.1010401)

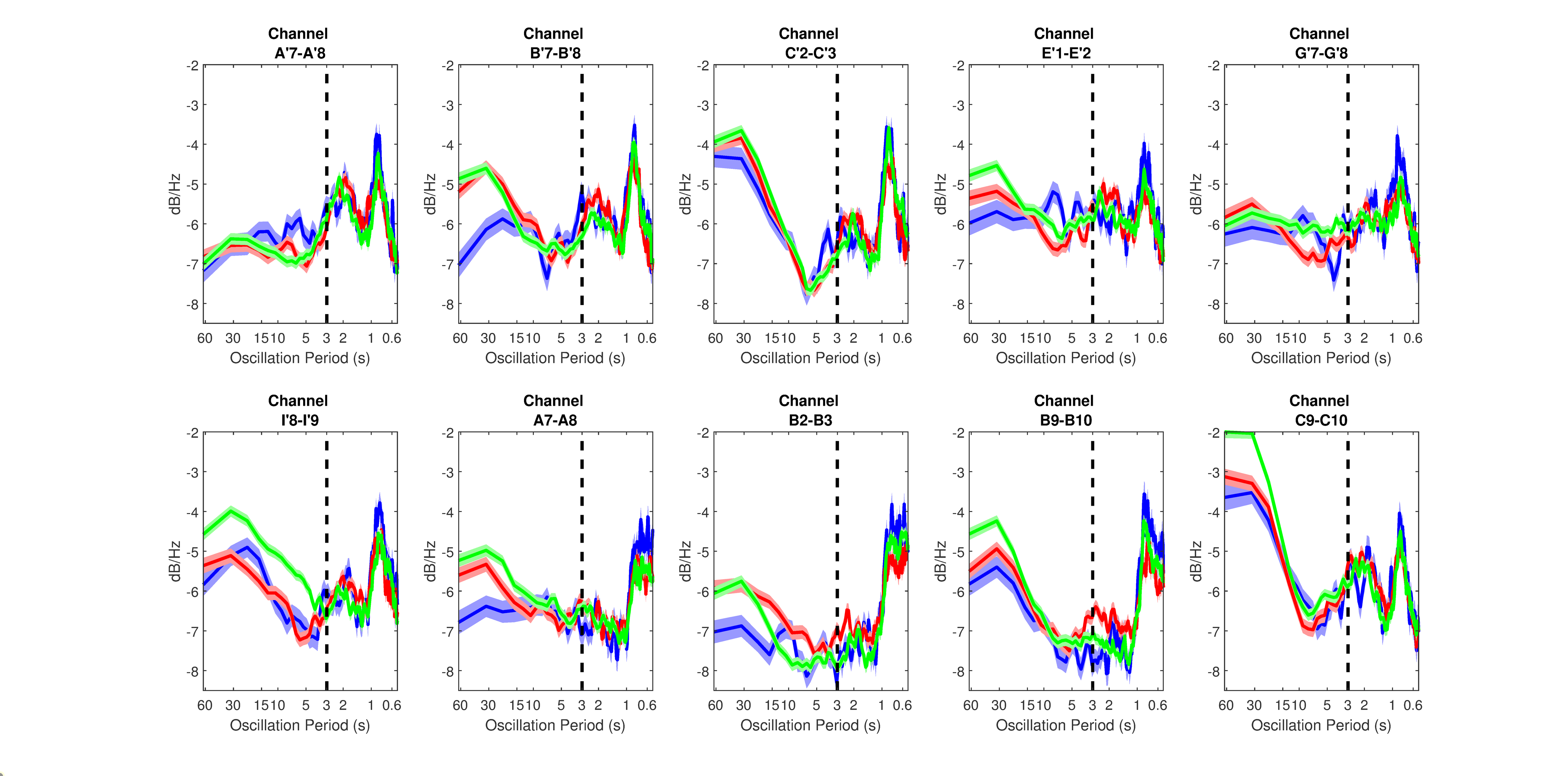

Supplement: S1 Fig — (TIF) [file pcbi.1010401.s001.tif]

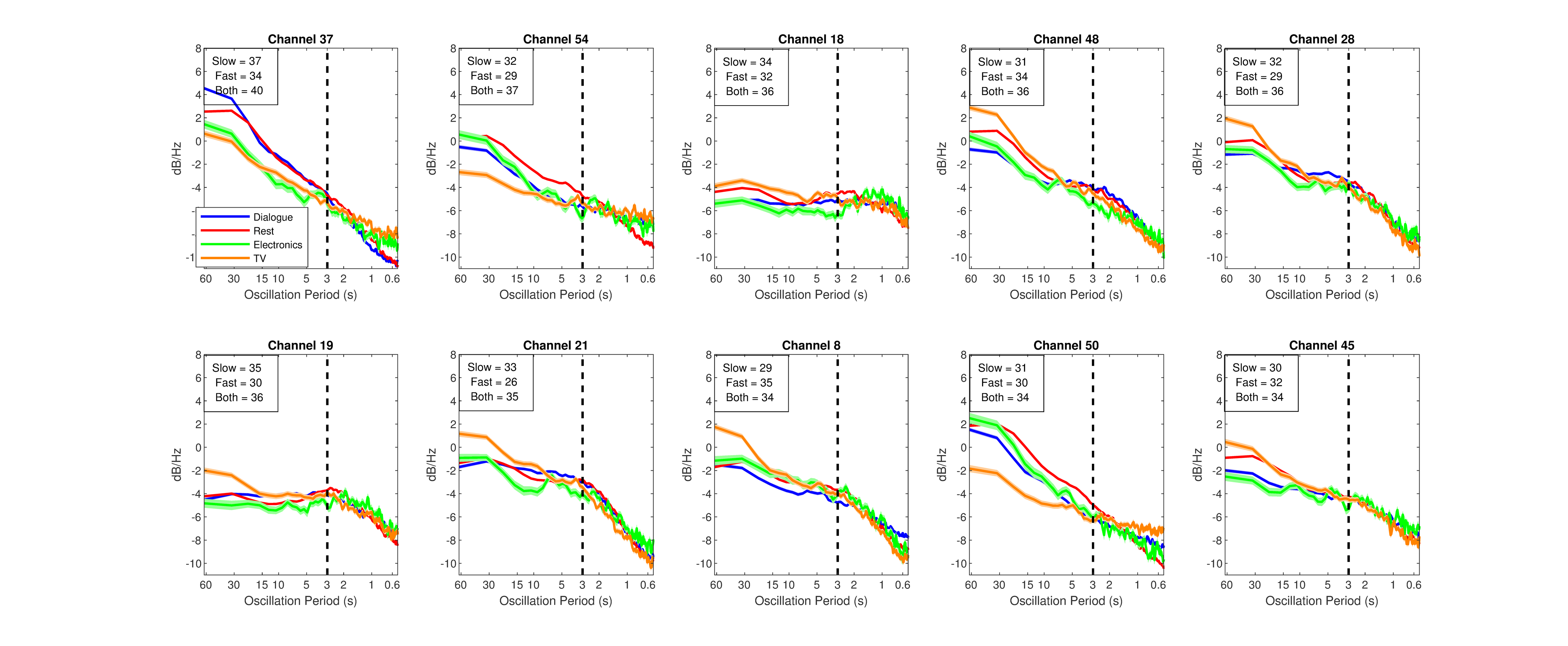

Supplement: S2 Fig — (TIF) [file pcbi.1010401.s002.tif]

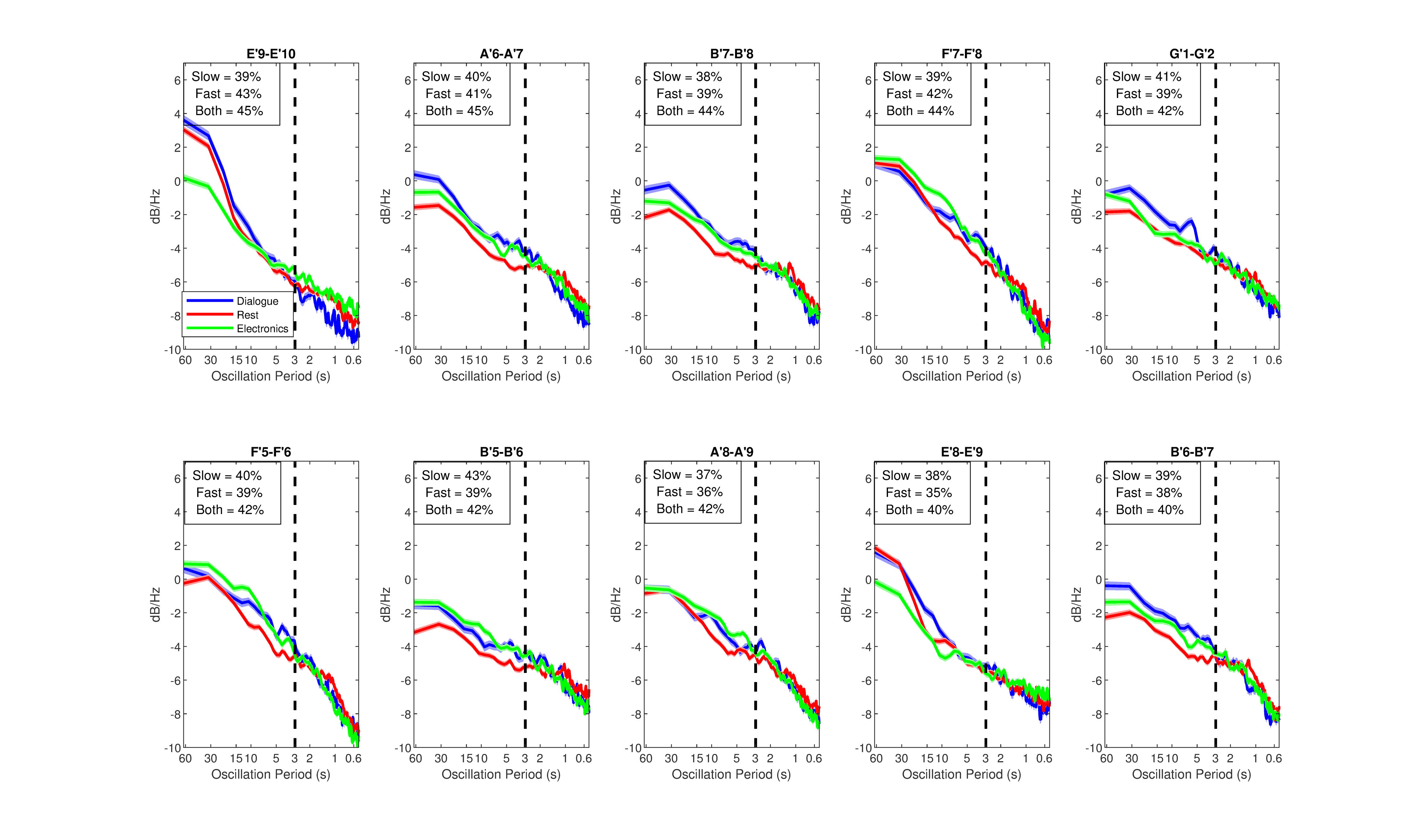

Supplement: S3 Fig — (TIF) [file pcbi.1010401.s003.tif]

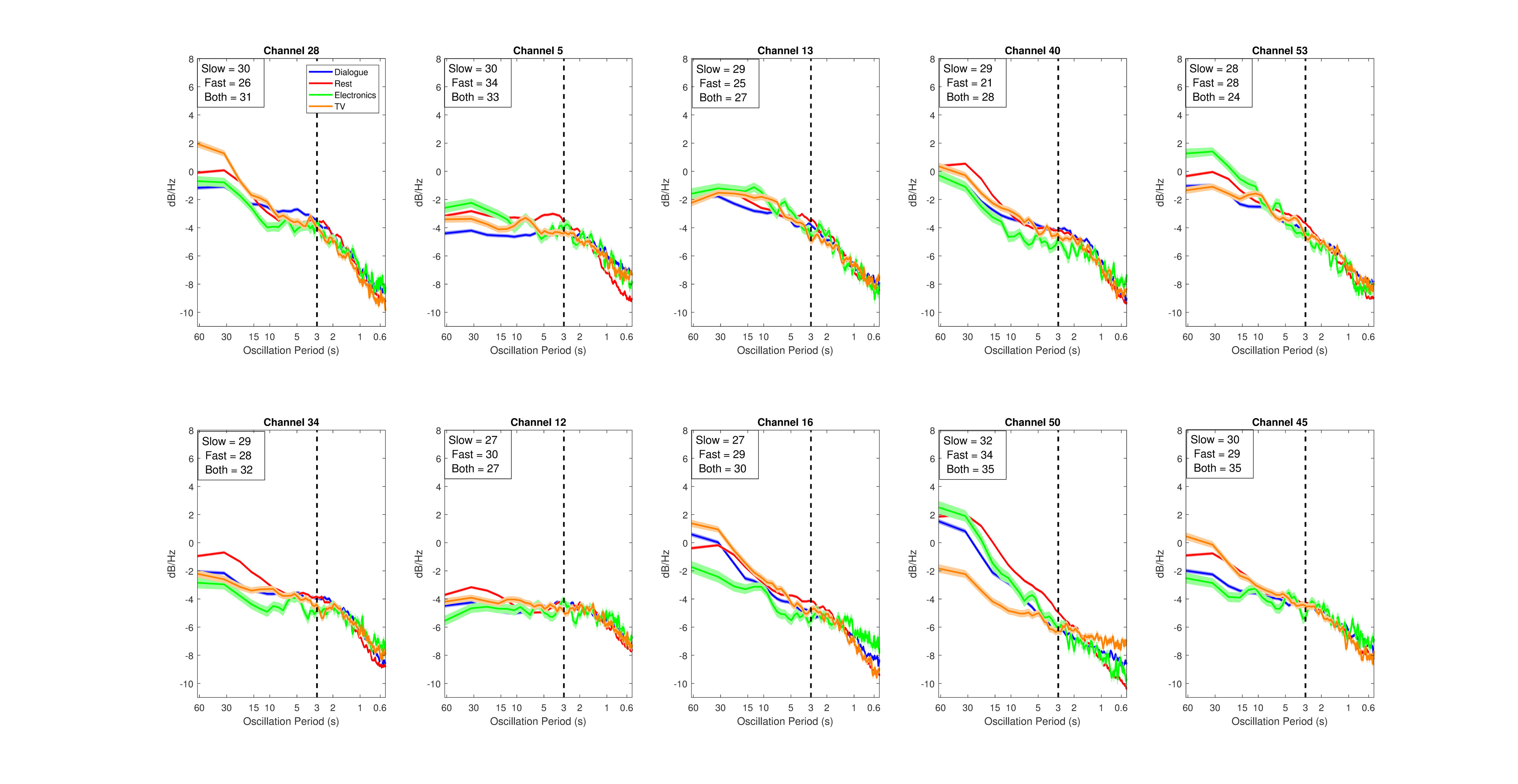

Supplement: S4 Fig — (TIF) [file pcbi.1010401.s004.tif]

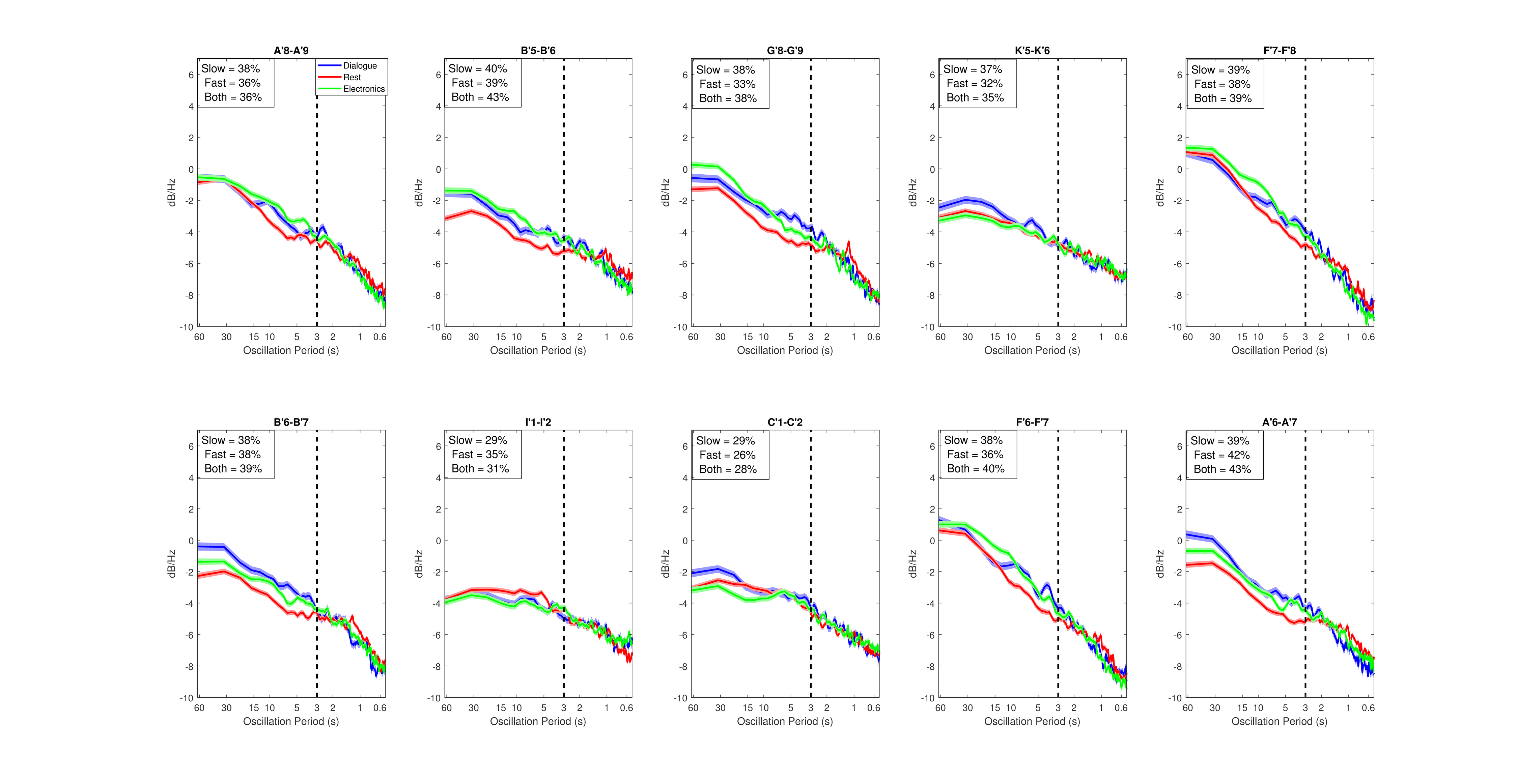

Supplement: S5 Fig — (TIF) [file pcbi.1010401.s005.tif]

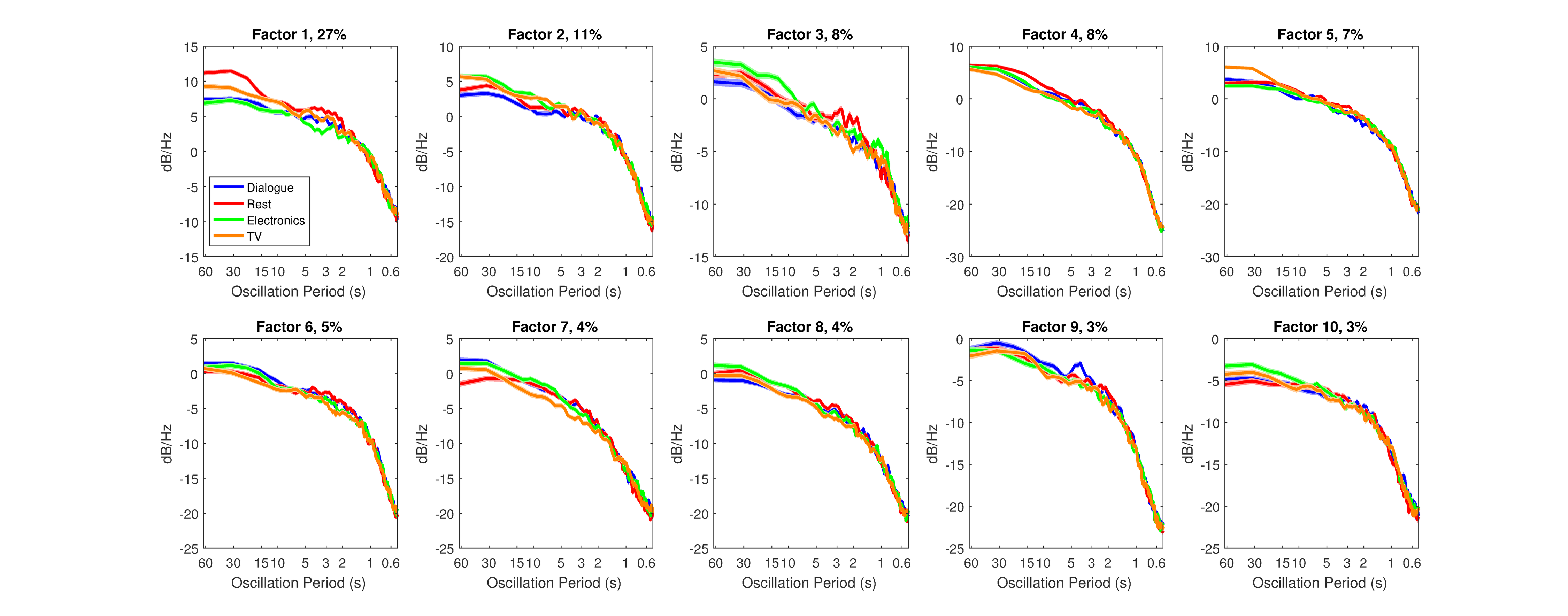

Supplement: S6 Fig — The percentage of variance explained for each factor is displayed in the title of each factor. (TIF) [file pcbi.1010401.s006.tif]

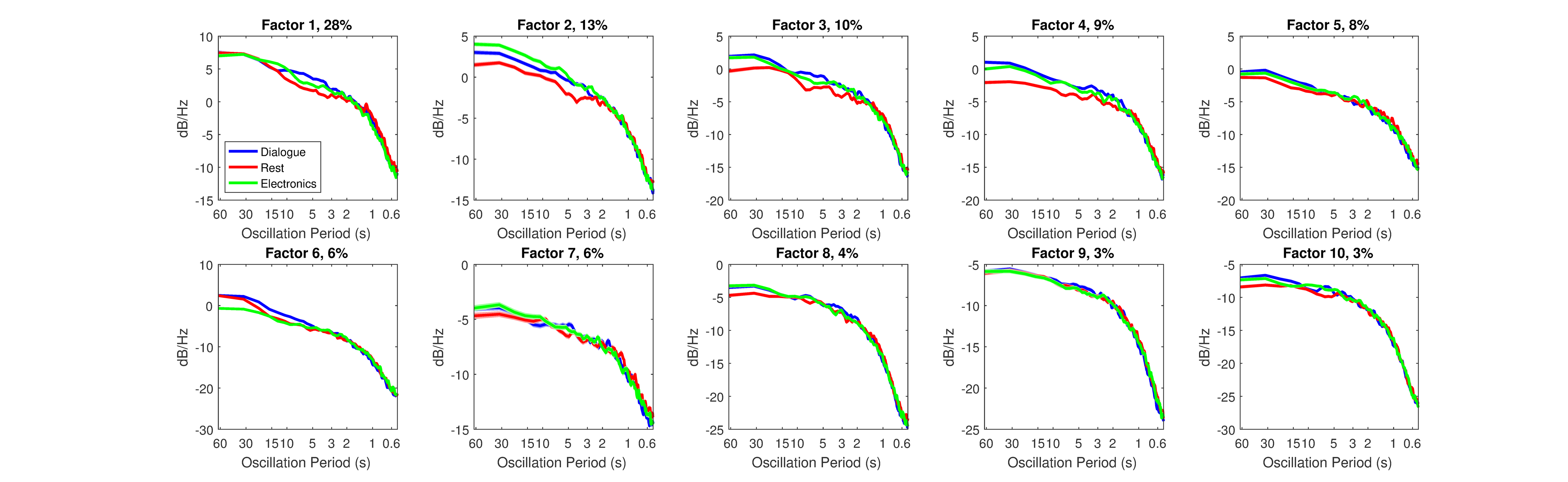

Supplement: S7 Fig — The percentage of variance explained for each factor is displayed in the title of each factor. (TIF) [file pcbi.1010401.s007.tif]

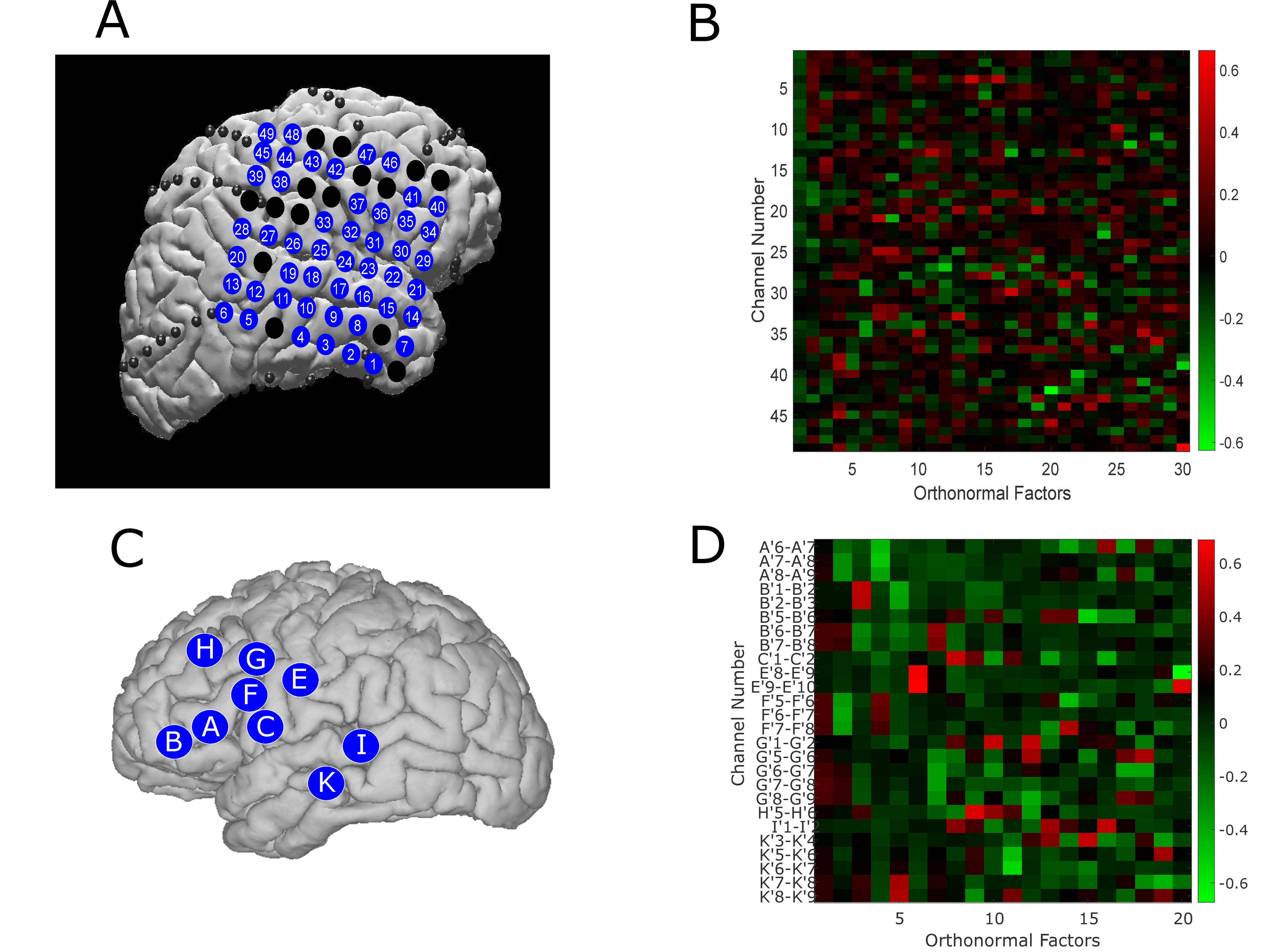

Supplement: S8 Fig — Locations of ECoG channels and sEEG shank entry points are shown for Subject 1 and 2 in subpanels A and C respectively. (TIF) [file pcbi.1010401.s008.tif]
