# Supplementary material for: Spatiotemporal dynamics of human high gamma discriminate naturalistic behavioral states
Source: PLoS Comput Biol. 2022 Aug 8;18(8):e1010401. doi: 10.1371/journal.pcbi.1010401 (PMC9387937; doi:10.1371/journal.pcbi.1010401)
Supplement: Attachment — Submitted filename: Reviewer Responses_V2.docx [file pcbi.1010401.s009.docx]

Dear Dr. Alasfour,

Thank you very much for submitting your manuscript "Spatiotemporal Dynamics of Human High Gamma Discriminate Naturalistic Behavioral States" for consideration at PLOS Computational Biology.

As with all papers reviewed by the journal, your manuscript was reviewed by members of the editorial board and by several independent reviewers. In light of the reviews (below this email), we would like to invite the resubmission of a significantly-revised version that takes into account the reviewers' comments.

Two reviewers think your paper has definitely merits, but it has some problems that need to be fixed, in particular it needs clarification on a number of issues. I encourage you to pay close attention to these suggestions and submit a revised version.

We cannot make any decision about publication until we have seen the revised manuscript and your response to the reviewers' comments. Your revised manuscript is also likely to be sent to reviewers for further evaluation.

When you are ready to resubmit, please upload the following:

[1] A letter containing a detailed list of your responses to the review comments and a description of the changes you have made in the manuscript. Please note while forming your response, if your article is accepted, you may have the opportunity to make the peer review history publicly available. The record will include editor decision letters (with reviews) and your responses to reviewer comments. If eligible, we will contact you to opt in or out.

[2] Two versions of the revised manuscript: one with either highlights or tracked changes denoting where the text has been changed; the other a clean version (uploaded as the manuscript file).

Important additional instructions are given below your reviewer comments.

Please prepare and submit your revised manuscript within 60 days. If you anticipate any delay, please let us know the expected resubmission date by replying to this email. Please note that revised manuscripts received after the 60-day due date may require evaluation and peer review similar to newly submitted manuscripts.

Thank you again for your submission. We hope that our editorial process has been constructive so far, and we welcome your feedback at any time. Please don't hesitate to contact us if you have any questions or comments.

Sincerely,

Marieke Karlijn van Vugt, PhD
Associate Editor
PLOS Computational Biology

Daniele Marinazzo
Deputy Editor
PLOS Computational Biology

***********************

Two reviewers think your paper has definitely merits, but it has some problems that need to be fixed, in particular it needs clarification on a number of issues. I encourage you to pay close attention to these suggestions and submit a revised version.

We thank the editor and reviewers for the time applied to our manuscript and for the thoughtful comments. Based upon the reviewers’ responses we have revised analyses and corresponding figures and have made edits to the manuscript text. Our detailed response to reviewer concerns are below and here we summarize the concerns raised and the revisions made:

1) Figures 2, 6, and 7 have been edited to display the finite chance performance derived from the cumulative binomial distribution.
2) For Figure 3, we included in the caption the location of the electrodes and referenced Figure 7.
3) We slightly changed our conclusions regarding Figure 7 since we are now comparing the single factor performance to the finite chance level. Figure 7 shows that only a subset of single spatio-temporal factors at certain timescales provide marginal information regarding behavioral states. Therefore decoding performance is most likely related to the a combination of spatio-temporal factors.
4) Added new references in the discussion regarding studies on the neural correlates of naturalistic behavior for both rats and humans.
5) We added Supplementary Figures 4 and 5 which show the power spectral densities of randomly selected channels for Subjects 1 and 2.
6) We clarified some our choices of the timescale parameters used in this study in the Methods Section (mainly our choice of 250 ms and 3 second bins).
7) We have referenced “Drew, Duyn, Golanov and Kleinfeld, 2008, in Nature Neuroscience News and Views” in the discussion. We have pointed out that the source of ultra-slow gamma band power modulations can have subcortical sources, and that these modulations are behavior specific. This point is highly relevant to our conclusions in this work.
8) We added text in the Discussion Section regarding the need for algorithms supervised algorithms that can incorporate unbalanced datasets.
9) As per the editorial staff request, we have made the data and code available and have edited our Data Availability statement to reflect that.

Reviewer's Responses to Questions

**Comments to the Authors:
Please note here if the review is uploaded as an attachment.**

Reviewer #1: This manuscript addresses the important question of how unstructured behavioral states are represented in neuronal population signals. ECoG and sEEG signals were measured from 2 human patients and high gamma band activity was extracted. The authors describe how high gamma band fluctuations at different frequencies may encode different behavioral states and the example in figure 1 clearly describes the problem. They show a novel approach for decoding bran states where slow and rapid timescales in high gamma band activity can separate behavioral states of watching TV, using electronics, engaging in dialogue and rest.

The estimation of the chance rate for the decoding performance seems to be set at .25 for subject 1 and .33 for subject 2. Simulations or the binomial formula should be used to better assess the classification results.

Thank you for pointing this out. We have edited Figures 1, 5, and 6 to incorporate binomial chance performance. There have not been any changes in our conclusions regarding Figures 1 and 5. However, in Figure 6 we note that now a smaller subset of factors exceed binomial chance performance. This indicates that individual factors when taken separately only contain a small amount of information that is behavior specific. However, when combining this knowledge with the results in Figure 5, we note that there certainly is ‘additive information.’ It seems that the interplay between different spatio-temporal features is behavior specific.

Previous studies have linked slow high gamma band dynamics measured in similar fashion to the fMRI signal, see for example: Drew, Duyn, Golanov and Kleinfeld, 2008, in Nature Neuroscience News and Views. It would be helpful to discuss the relation between the timescales found in the current study and this type of work.

We have briefly mentioned that slow changes in high gamma band power are related to information accumulation according to Nir et al (Interhemispheric correlations of slow spontaneous neuronal fluctuations revealed in human sensory cortex. Nature Neuroscience. 2008). However, we will elaborate more in the text.

Clarification should be provided for choosing 3 secs to separate slow from fast high gamma band dynamics.

We have provided the clarification in the text. Our previous work has shown that we can classify coarse behavioral states using 3 seconds as our temporal resolution, so we choose this time resolution to connect to this previous applied work. We view 0.333 Hz as an appropriate cutoff frequency to distinguish between fast and slow high gamma band dynamics.

The paper provides a nice theoretical framework for decoding brain states, but the link between different networks, timescales and factors is still a bit unclear. For example, if the factors in Figure 8 would be removed from the data, like a virtual lesion, would the same decoding still be feasible? This would help the reader understand whether a certain factor arising from a network is ‘essential’ for the approach.

A virtual lesion analysis is a powerful tool to identify whether a certain factor is critical for classification. However, we believe that Figures 5 and 6 strongly suggest that no single factor dominates classification accuracy:

A) Figure 5 demonstrates that factors contain ‘additive information.’ If one factor is ‘essential’ we would likely see a strong jump in classification accuracy and then a flat curve. Figure 5 also shows that starting from fast factors or slow factors both show a monotonic increase in performance as a function of the number of factors.
B) Figure 6 demonstrates that only a subset of factors contain information regarding behavioral context, and the amount of information that each factor individually contains regarding behavioral context does not indicate that one specific factor is essential.

Your comment’s merit is definitely taken into account and we believe that our results indicate that there is an additive information from different spatio-temporal features that are behavior specific. We ran your suggested analysis and found that applying a virtual lesion using any of the extracted factors did not decrease performance compared to using all of the factors. In this work, we think it is best to not include a figure for the virtual lesion analysis. The reason being is that since we generate new set of factors in every training iteration, it is difficult to make precise conclusions regarding the effects of a specific spatio-temporal factor on decoding performance. Additionally, we downsample the training set and therefore the spatio-temporal factors extracted do not take advantage of the full power of the data. We aim that this work to serve as an exploratory study where the most important result is that spatio-temporal factors (after removing the effects of long-term mean and variance) do separate naturalistic behavioral states. We have mentioned in the discussion the need for algorithms that can help with the analysis of specific spatiotemporal factors from naturalistic class-unbalanced datasets.

Minor comments.
The scale on the y-axis differs between Figure 6A and 6B, similar axes would allow for better comparisons across subjects. It units of tau should also be stated on the axis.

Thank you for this comment. We have set the limits to be identical and included a line to indicate binomial chance performance. We have also set the tau parameter to have the same limits for both subjects to highlight the differences in the temporal dynamics.

There was a typo in line 180: implanets
Line 225: “The covariance matrix across 30-sec epochs were” should be matrices?

You are correct, we have amended the mistake in the text.

Reviewer #2: Alasfour et al. describe an analysis of naturalistic ECoG and sEEG recordings, where they show that it is possible to classify coarse behavioral states from properties of these recordings. This is an interesting dataset, and the paper is a new take on decoding behavioral states, using both engineered features and GPFA. My more specific comments are as below.


- The mean and covariance calculations were done using 30-sec or 250-ms windows. How were these parameters chosen and justified, and how would any of the results change if different decisions were made with these hyper-parameters?

Thank you for the comment, we have edited the text in the manuscript to provide more detail on how these parameters were chosen.

Briefly, 250 ms was chosen based upon previous studies as an appropriate temporal resolution that can distinguish motor movement and visual stimuli, and practical considerations have been taken since increasing the temporal resolution (reducing the time window) would introduce noise and increase computation time when running further analyses.

Secondly, 30 seconds was chosen to capture enough neural activity such that fluctuations of high gamma band power that are less than 0.1 Hz are represented. These ‘slow’ fluctuations are shown by previous studies to be related to information accumulation and interhemispheric correlations.

- I don't find Figure 1 to be particularly helpful in the narrative of this very interesting paper. That it is possible for the covariance between multiple electrodes to change in a synthetic way that is discriminable, in simulation, is true. Perhaps this point can be better made if the real data in Fig 2 were visualized to show that it is indeed these second-order statistics in the data that are present in the data?

Thank you for this positive assessment of our paper. Our goal is to be accessible for a wide audience. Indeed, our lab debated whether Figure 1 was necessary. Ultimately, we found that Figure 1 was didactic when presenting the work to colleagues in our lab and in the broader research community. Figure 1 serves as an instructional figure that highlights the possible sources of variability in neural data. It would be very difficult to find a specific epoch of real data that clearly highlight ALL of the sources of variability that we discussed in this work. Please also see the comments from Reviewer 1 as their comments indicate that they also found Figure 1 to be helpful.

- How are the error shade plots in Fig 3 computed? Where in the brain are these two example electrodes? In other words, do the rest of the electrodes have the magnitude of the differences show here (beyond the biggest ones show in the supplement), and where in the brain are these electrodes located?

The error plots were computed from the power spectral density calculation prior to averaging all the periodograms. The standard error is shown in Figure 3. Thank you for pointing out this potential source of confusion. We now provide additional clarification in the text. For Subject 1 the example electrode is channel number 37. For Subject 2, the example electrode is the bipolar pair E’9-E’10. Please refer to Figure 7 to locate where these electrodes are located in the brain. We now refer the reader to Figure 7 in the text as well.

We have added a supplemental figure showing a randomly subsampled set of electrodes for both subjects. Some electrodes do not show discriminability in the PSD that is related to behavioral state.

- I'm a little confused about the 3 subjects in this paper. Many of the results show only 2 subjects?

We have mentioned in the text that one subject has been excluded from further analysis due to the presence of high frequency noise. Supplementary Figure 1 elaborates on this issue.

- How does the GPFA factor decoding compare to the first- and second-order statistics decoder? By comparing the plots, they look like they are at least in the same ballpark. Is that true quantitatively? And either way, might the authors discuss the relative merits of the different approaches to decoding?

Thank you for asking this question as we did not explicitly state the accuracy using the GPFA decoder when incorporating all of the factors. The performance of the decoder using all of the GPFA factors are 71% and 61% for Subject 1 and 2 respectively. This is comparable to the performance using first and second order statistics.

Also, regarding to the relative merits. It is difficult to ascertain the merits of each feature set. However, one can hypothesize that first and second order statistics might be less correlated to complex behaviors and more correlated to the general state of the brain.

, the authors may be interested to know about a recently published dataset, AJILE12 (Peterson et al.), which has similar ECoG data and some behavioral labels (e.g. talking, watching TV/computer) for 12 participants. It would be interesting to see if the analyses the authors presented here would generalize to another dataset with more subjects!

We are aware of the work done from the Brunton lab and the new AJILE12 dataset that has been recently published. In the discussion we now mention this new work and elaborate that the next step is to investigate whether our results generalize to more subjects and data!
